# Historical information sheds new light on the intensification of flooding in the Central Mediterranean

**DOI:** 10.1038/s41598-023-37683-z

**Published:** 2023-07-01

**Authors:** Nazzareno Diodato, Fredrik Charpentier Ljungqvist, Gianni Bellocchi

**Affiliations:** 1 Met European Research Observatory – International Affiliates Program of the University Corporation for Atmospheric Research, Benevento, Italy; 2 Department of History, Stockholm University, 106 91 Stockholm, Sweden; 3 Bolin Centre for Climate Research, Stockholm University, 106 91 Stockholm, Sweden; 4 Swedish Collegium for Advanced Study, Linneanum, Thunbergsvägen 2, 752 38 Uppsala, Sweden; 5 Université Clermont Auvergne, INRAE, VetAgro Sup, UREP, Clermont-Ferrand, France

**Keywords:** Climate sciences, Environmental sciences, Environmental social sciences

## Abstract

Hydrological disasters, such as floods, can have dire consequences for human societies. Historical information plays a key role in detecting whether particular types of hydrological disasters have increased in frequency and/or magnitude and, if so, they are more likely attributable to natural or human-induced climatic and other environmental changes. The identification of regions with similar flood conditions is essential for the analysis of regional flooding regimes. To this end, we here present the longest existing flood reconstruction for the Eastern Liguria Area (ELA) in northwestern Italy, covering 1582 to 2022 CE, which offers a case study representative of the central Mediterranean region. An Annual Flood Intensification Index was developed to transform the historical data into a continuous annual hydrological time-series contained by a homogeneous data structure for the study-area. We found two change-points (trend breaks) in the reconstructed time-series, in 1787 and 1967, with only occasional heavy floods comparable to present-day disasters occurring before the first change-point, and an increasing intensification of floods after the second change-point up to the present day. The recent intensification of flooding in the ELA, associated with changes in land use and land cover, also appears to coincide with phases in which hydrological hazards have become more changeable and extreme in disaster-affected areas. This is evidenced by river basin responses to human-induced disturbances.

## Introduction

Landscape features support a range of ecosystems, influence hydrological processes and streamflow responses to climate change, and control feedback mechanisms between water, energy and ecological processes^1,2^. In some parts of the world, changes in land use and extreme events are increasingly affecting such hydrological processes and responses^3^. However, despite recent observed increases in precipitation extremes in parts of the world, there is still little evidence of systematic increases in either flood magnitude or frequency compared to historical baseline conditions^4^. This is partly due to the fact that flooding is closely linked to landscape sensitivity conditions, which are more difficult to identify and which cause them to change in space and time. Temporal sensitivity reflects the *strength* and *frequency* of single storm events nested within patterns of longer-term environmental changes occurring on different timescales^5^. However, approaches to detect flood variability from historical climatology are scarce, although they are critical for understanding landscape sensitivity to past environmental changes. Here we can recall events studied in some detail by reconstructing multi-decadal and multi-centennial flood time-series at global^6^, continental^7,8^, regional^9,10^ and local^11^ geographical scales. Floods have thus been widely used by climate historians as a possible indicator of long-term climate change^12,13^.

**Figure 1:**
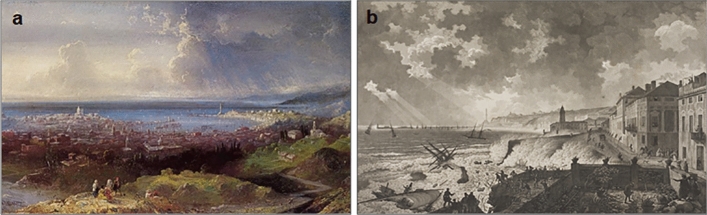
Images of the city of Genoa in the mid-nineteenth century. (**a**) View of the city under a storm by the Swiss painter and reporter Carlo Bossoli (1815–1884) from 1872 (from: Musei di Strada Nuova – Palazzo Bianco, Genova, Italy: https://www.museidigenova.it/it/veduta-di-genova-1872); (**b**) Aquatint by the Italian painter Luigi Garibbo (1782–1869) portraying the scene of the storm that hit Genoa on 21 December 1821 from the seafront road east of the church of San Teodoro, from where the arch of the port can be seen to the fortifications of San Benigno, the lantern and the new pier (from: Musei di Strada Nuova—Palazzo Bianco, Genova, Italy: https://www.museidigenova.it/it/veduta-del-porto-di-genova-durante-la-tempesta-nei-pressi-di-s-teodoro).

Landscape responses to changing disturbance regimes are increasingly likely to be influenced by the linkage of past damaging hydrological events, as the time-span between successive disturbances decreases whereas the frequency of flooding increases^14,15^. Then, thunderstorms following days of continuous rain can alter the hydrogeological and hydraulic conditions of a landscape. This is the case of Eastern Liguria Area (ELA) in Italy, the focus of this study, where rainfall can be heavy for many days or months (not every day in a month), or falls violently for days at a time. Accordingly, the streams, descending from the upper parts of their basins, gradually receiving the inputs from the tributaries, flow into the terminal strip of their course, usually flat, and reached their natural destination, the sea (Fig. 1a).

The Italian geologist and statesman Lorenzo Pareto (1800–1865) wrote, illustrating the characteristics of the Genoa area at the beginning of the nineteenth century (Pareto^16^, p. 112):

*[…] those of short course and humble torrents, which descend rather impetuously from steep slopes in copious rains, but do not expand to large floods in overflowing the banks, returning shortly to the natural boundary* [our translation].

The environmental conditions that support the landscape in its ecological-social aspects are also important. These conditions can vary from one time to another, with flooding events which in turn can be exacerbated or mitigated by the degree of resilience of the landscape. For instance, the environmental and economic impact of these phenomena have also been high in historical times. The study of the most famous fluvial floods suffered by the human population since the beginning of the modern era offers an opportunity to conceptually overcome an overly strict distinction between natural and man-made disasters. Contemporaries perceived the occurrence of such phenomena as the effect of both nature and human causes. Disasters of this magnitude to the detriment of cities left a lasting impression on the people of the time. Reporters, ecclesiastical and government officials left substantial documentation of some of them, including both narratives and legislative provisions. Streets and squares were inundated and damaged by extraordinary floods, as Monsignor Agostino Giustiniani (1470–1536) reported in his *Castigatissimi Annali di Genova* (Pareto^16^, p. 157):

*Our stories mention some rain showers that caused very serious damage to our city; the most memorable is that of 1278 reported by Giustiniani. On 8 October of that year, there was such a deluge of water that it rose 10 palms over the Piazza di Banchi. The rains of 1407, October 31 and 1414, October 3 were also very terrible: in that of 1407 the waters flowed 6 feet high in various streets of the city and ruined several houses and walls* [our translation].

However, the southern-most regions of Europe are particularly vulnerable to erratic storms that are in a continuous and dynamic interaction with the landscape, sometimes making it difficult to distinguish the cause from the effect of flooding. This is how the Italian journalist Davide Bertolotti (1784–1860) describes the terrible event of December 1821 affecting the city of Genoa (Bertolotti^17^, p. 192; Fig. 1b):

*A storm of mournful memory raged in the Gulf of Genoa on the night of 24 to 25 December 1821, and continued until the 27*^*th*^*. Carried, or rather hurled, by the furious south-westerly wind, the waves rose up to the roofs of the houses located on the sea to the west of the church of the Graces. The whole port was covered with shipwrecks* [our translation]*.*

At the same time, several areas in recent decades have witnessed an increase in daily and/or hourly precipitation extremes in Europe and globally, leading to an increase in flood risk^18–20^, and that in Italy itself, extreme rainfall trends in the ELA have been increasing scatteredly for certain durations of 3–24 h, during the period 1940–2015^21^. The ELA is rich in historical documentary data dating back to the sixteenth century. Historical documentary data from regions with long-standing archives provide a unique opportunity to reconstruct past climate events on a daily basis with accurate dating^22^. In order to better understand the variability and changes of diluvial water and floods, as well as their effects on ecosystem functioning and societal impacts, reconstructions from such documentary archives of past precipitation extremes are crucial^23^.

Recent research has focused more broadly on historical climatology^24–26^, as well as on the reconstruction and impacts of extreme precipitation events. For instance, the Past Global Changes (PAGES) Floods Working Group has begun multidisciplinary analyses of historical floods and their impacts using a variety of archival proxies from natural and documentary archives^27,28^. In recent years, there has also been new works on reconstructing past storms^29,30^. Studies of past climate in Europe^31–35^ have revealed a long history of wetter and drier periods, with regular and often sudden cycles, which have caused devastating floods^9,36,37^. However, the occurrence of extreme weather events, such as torrential rainfall and associated floods, is difficult to record, as such events are highly dependent on season, location and geographical extent^7,10,11^. Furthermore, historical information may be affected by incompleteness in some flooding classes. To overcome these difficulties, in this study we have developed a new approach that has been applied to eastern Liguria.

Due to the geo-morphological and geo-hydrological characteristics of its territory, with coastal cities that do not extend inland, Liguria is exposed to natural phenomena that are potentially dangerous for people and things. In fact, the history of the region is dotted with destructive events that, over time, have caused huge damage and numerous casualties. Several authors have studied geo-hydrological hazard and its recent increase by analysing meteorological and climatic factors and their interaction with the complex orography of Genoa and surrounding localities^38,39^. The fragility of the Ligurian territory is not a recent phenomenon, but already in historical times, according to previous testimonies, and by recent works for the Bisagno basin^40,41^, recurrent floods are documented, fairly regularly from the nineteenth century^41^. The fluvial processes originating in the Entella basin were, instead, studied by Roccati et al.^42^, who presented a list of the main floods from 1626 to 2016.

**Figure 2:**
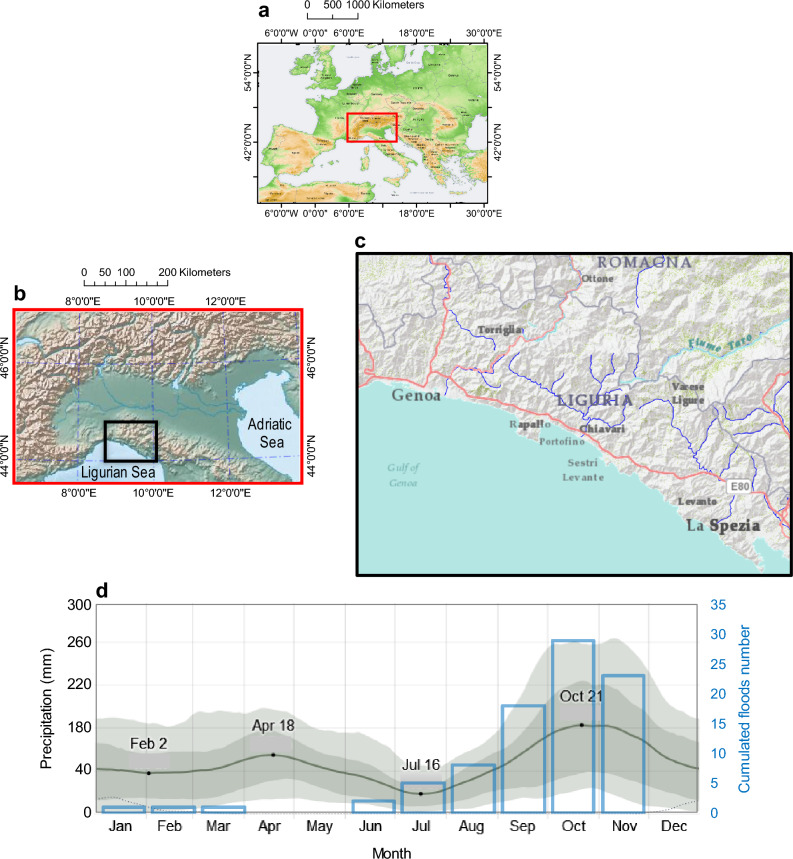
Environmental setting. (**a**) Map of the study region (from ISPRA-SCIA: http://193.206.192.214/servertsutm/serietemporali400.php ); (**b**) The Eastern Liguria Area (bounded by the black line), with the relief of northern Italy (from ISPRA-SCIA: http://193.206.192.214/servertsutm/serietemporali400.php); (**c**) Eastern Liguria Area, with the main basins; (**d**) Mean monthly precipitation over the period 1981–2012, with the mean rainfall (solid black line) accumulated over the course of a 31-day sliding period centred on the day in question, with 25th to 75th and 10th to 90th percentiles (grey bands) (from Weather Spark: https://weatherspark.com); blue bars are the monthly floods that occurred in the period 1850–2022.

The current study presents the first monthly-resolved reconstruction of flood events occurred in the ELA over the 1580–2022 CE period. The landscape of the ELA extends from Genoa to the mouth of the Magra River in the province of La Spezia (Fig. 2a,b). The region is almost entirely hilly and mountainous, with many inaccessible zones. The only plains worth mentioning are the Piana dell’Entella, behind the cities of Chiavari and Lavagna, part of the territory of Sestri Levante and the area around the mouth of the river Magra, on the border with Tuscany. In the eighteenth century, Cardinal Filippo Casoni (1733–1811) of the Roman Catholic Church praised the beautiful landscape of eastern Genoa with these words (Quaini^43^, p. 205):

*Leaving Genoa to the east, after a few beaches and hills full of sumptuous palaces and delightful villas, one discovers the plains of Quinto and Nervi, in respect to which nature and art are fully interested in making that happy situation a continuous very pleasant receptacle for rural delights* [our translation]*.*

The main hydrographic basins included in the ELA are the Polcevera (140 km^2^), the Bisagno (95 km^2^), the Magra (1270 km^2^), the Lavagna (160 km^2^) and its tributary Entella (Fig. 2c). I﻿n the ELA, the main watersheds and the most important reliefs remain at high altitudes, but lower than those present in the extreme western sector of the Ligurian Alps. Morphology has a strong influence on the climatic characteristics of Liguria, since the slopes, valleys and ridges have such altitudes, orientations and distances from the Ligurian Sea that they influence the horizontal and vertical direction of the atmospheric currents that flow into it. In particular, the direction of the river valleys, some almost meridian, such as those of Roia and Polcevera, others longitudinal, such as those of Arroscia, Lavagna and Vara, change the direction of the movement of atmospheric flows close to the ground^44^. Here, two main elements contribute to the formation of heavy precipitation: a high water vapour content in the atmosphere and events triggered by thermodynamic or dynamical processes^45^. On their way to warmer waters, Mediterranean cyclones form mainly around a few centres, with the dominant region in the Gulf of Genoa, where a slow-moving trough (or Vb weather pattern) can bring large amounts of precipitation^46^. Precipitation in the ELA almost never drops below 1100 mm year^−1^, with an annual mean in Genoa of 1153 mm year^−1^ during the period 1981–2016. Interannual variability is also high, and on the Genoa coast it can occasionally exceed 1400 mm year^−1^ (75th percentile), while the maximum is 2765 mm year^−1^, which occurred in 1872 (data obtained from the European Climate Assessment & Dataset project; http://climexp.knmi.nl).

Due to the geo-hydrological risk and uncontrolled building, the ELA in general, and the Genoa metropolitan area in particular, represent a case study of interest for the central Mediterranean^47^. For the ELA, we provide a continuous time-series of annual flood-causing storms that affected riparian cities and inland villages in the period 1582–2022 CE. We developed an Annual Flood Intensification Index (AFInIx) based on a systematic and critical analysis of data from ELA documentary sources on the above-mentioned phenomena. In order to understand how changing climatic patterns, including those that may be related to recent global warming, affect the temporal variability of heavy storm events and flooding episodes, reliable monthly reconstructions of flood occurrence are crucial^10^. Hydrological reconstructions, regardless of the time-scale employed, also allow assessment of their impact on ecosystem functioning and economic security at the regional and local scales^48,49^. In most cases it was possible to verify the events using more than one documentary source. It was also possible to place the storm events in the context of other types of historical events (i.e., economic, social, agricultural and religious). The major difficulties stem from the lack of continuously collected observations, which would allow the recurrence of these processes to be determined. To overcome this difficulty, AFInIx was designed to compare the scale-invariance in the relationship between the number of events above the intensification strength and events of the same strength, i.e., to test the completeness of the catalogue of extremes for the entire dataset from 1582 to 2022 CE. In this way, the historical data served to obtain an informative historical time-series on the current state of the area and on the evolutionary dynamics spanning a wide time-interval.

## Results and discussion

### Completeness of catalogue data and flood hazard index estimation

Various types of historical documents have been utilised to gain insights into the rich history of the ELA and its experiences with floods and alluvial events. As reported in Supplementary Table S1, these include annals and chronicles providing narratives of significant events, official records offering governmental reports and administrative documents, statistical publications providing data on floods and precipitation, geographical and topographical works describing the region’s physical characteristics, archaeological studies revealing the historical development of rivers and structures, and scientific publications contributing to understanding past climate events and their impact. These diverse categories of historical documents contribute together to a comprehensive understanding of floods and alluvial events in the region. Since only a few floods were reported in the documentary sources with a detailed description of the events (generally only those of a certain severity), it was not possible to assign a damage indicator to each event in the entire flood catalogue. Thus, in order to create a homogeneous database that provides a continuous list of flood damage for the entire catalogue, we devised a statistical approach that took into account an objective criterion. To this end, a model function was assigned to each month in order to take into account floods of different magnitude between years. This function is referred to here as the *Annual Flood Intensification Index* (AFInIx).

**Figure 3:**
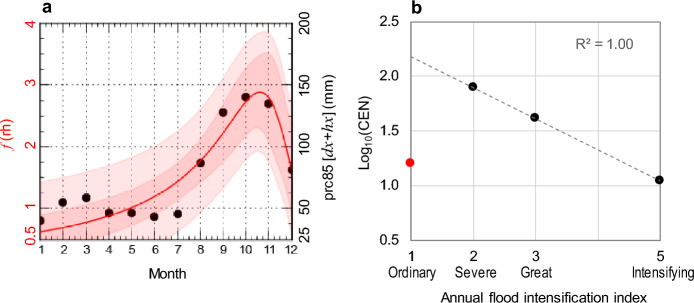
Precipitation and flood regimes. (**a**) Monthly regime of the 85th percentile (*prc85*) of the sum of daily (*dx*) and hourly (*hx*) maximum monthly rainfall (black dots), and Rationale Model function, *f*(*rh*), for flood intensification recognition (red line) during the period 2005–2021 (Supplementary Table S1); (**b**) Cumulative distribution of the logarithm of the number of flood events versus their Annual Flood Damage Index (AFDI) in the ELA during the period 1582–2022 CE. The magnitude 1 of AFInIx (red dot) is not aligned around the dashed line, so this type of event is considered incomplete in the catalogue and is deleted from the time-series to homogenise the catalogue.

In order to see how this function might be climatologically appropriate to distinguish the greatest impact in each month, we estimated the 85th percentile (*prc85*) of the sum of observed monthly maximum daily (*dx*) and hourly (*hx*) rainfall (mm) between 2005 and 2021, represented a*s prc85*(*dx* + *hx*) on the right-hand axis of Fig. 3a (where a multiplier of 0.02 ensures *f*(*rh*) = prc85(*dx* + *hx*) on an intensity scale varying between 0.5 and 4.0), and constrained these observations with respect to the weight function, so that we could solve for the most appropriate coefficients. The Rationale Model function *f*(*rh*), as similarly addressed in previous studies^30^, is able to modulate the hourly intensity of intra-monthly precipitation and can be used as a proxy for the recognition of flood intensification (Fig. 3a, red line). As can be seen in Fig. 3a, the red line describes a factor of the function that is maximal in the autumn months, when flooding is associated with multiple hydrological events (e.g., floods, accelerated erosion, landslides, bank erosion). This becomes important for the purpose of the weight that the function attributes to each individual event. Thus, to obtain a more comparable AFnIx between years, we multiplied the number of floods per year (*NFy*) by the monthly function model (*f*(*rh*):

1 $$\mathrm{AFInIx}=NFy\cdot f\left(rh\right),$$

2 $$f\left(rh\right)=\frac{a+b\cdot j}{1+c\cdot j+d\cdot {j}^{2}}\mathrm{with }j = 1 \left(\mathrm{January}\right), \dots , 12 \left(\mathrm{December}\right),$$

where *f*(*rh*) is the Rationale Model function introduced above. Working on a monthly basis, this function makes it possible to differentiate the intensity of an autumn storm from a winter or spring storm and, in turn, the effects of a flood in one year and not in another. The optimised parameters of the function *f*(*rh*) are (with in brackets the standard error): *a* = 0.5747 (± 0.114),* b* = − 0.0438 (± 0.0114), *c* = − 0.1664 (± 0.0075), and *d* = 0.00718 (± 0.001). The chosen function is better suited for representing a longer dataset compared to the limited time range of 2005–2011. This range primarily reflects recent warming trends and the resulting warmer winters in the Mediterranean region. These warmer winters have led to an increase in flooding events, predominantly occurring between February and March. In contrast, during colder periods in the past, winters experienced less intense precipitation and lower flood frequencies. To account for the long-term trend and the attenuated intensification of flooding events, we utilised the Rationale Model function (red line in Fig. 3a). This function offers improved interpolation of the long-term trend and allows for the recognition of the historical pattern in flooding events. Additionally, autumn has consistently experienced sustained extreme events both in the present and in the past, which the function accurately captures. Moreover, the function provides uncertainty bands that encompass the discrete values. It is noteworthy that all data points fall within the 90% uncertainty band, indicating the reliability of the function in representing historical variability.

As a result, we discovered the presence of 147 AFInIx episodes in the historical documentary data examined. Recording these events by hazard class, we obtained 16 ordinary events (bottomland overflow), 79 severe events (damage to crops and infrastructure), 41 great events (death of people and animals) and 11 intensifying events (rapid spread with multiple flood events). However, there may be several uncertainty factors in the designation of storm data. It is well known that historical documentary data tend to understate small isolated storms, especially when floods occur in areas with poor communications infrastructure^50^. To address some of these uncertainties, we developed a “reasonable criterion” for the recorded AFInIx. This was achieved by confirming the scale-invariance in the relationship between the number of events greater than flood-strength events and events of the same strength, or completeness analysis^51^. The latter was formalised by the relationship between the number of cumulative events (*CEN*) and AFInIx values within the range 1 ≤ AFInIx ≤ 5, as follows^52^: ${log}_{10}\left({CEN}_{i}\right)=a+b\cdot {\mathrm{AFInIx}}_{i}$, with *i* = 1, …, 5, where severity classes. The negative slope (*b*) in Fig. 3b represents the beginning of a downward trend as floods become larger.

With a coefficient of determination R^2^ = 1, the flood events from 1582 to 2022 CE can be assumed to be significantly scale-invariant and meet the criterion only for the 131 events that are described in qualitative terms as severe, great and intensifying floods within the range 2 ≤ AFDI ≤ 5. The remaining 16 AFDI = 1 floods do not fit the regression line shown in Fig. 3b (red dot in the scatter-plot).

Their number is much lower than that required by Eq. (1), most likely because many of these less energetic floods went unnoticed in the past. Events with AFInIx = 1, classified as ordinary floods, were excluded from the temporal analysis because they did not represent the entire catalogue from 1582 to 2022 CE. It should be noted that only floods caused by storms are considered in this study, so floods caused by snowmelt are not included in this catalogue.

### Temporal evolution of the reconstructed Annual Flood Intensification Index

**Figure 4:**
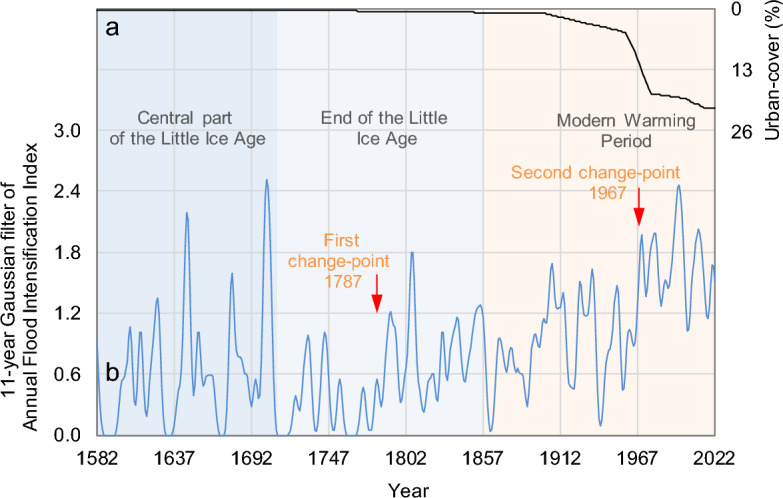
Eastern Liguria Area. (**a**) Evolution at multi-decadal scale of urban-cover in the Rapallo area^65^; (**b**) Temporal dynamics of 11-year Gaussian filter of Annual Flood Intensification Index (blue line) during the period 1582–2022 CE, with climate sub-periods indicated and the change-points at years 1787 and 1967 (red arrows).

In relatively small and mountainous basins, such as those composing the ELA, hydrological and geomorphological processes are characterised by nonlinear interactions between climatic constraints, land surface and fluvial responses on different spatial and temporal scales^53^. As pointed out by Mulligan and Wainwright^54^, these hydrological processes are strongly dominated by the spatial connectivity of runoff-producing elements. With the help of the historical sequence of stormy seasons, it is possible to summarise the influences of climate variability on floods during the period 1582–2022 CE. To identify possible trends and oscillations in the discrete data, the time-series was filtered using a 11-year low-pass Gaussian function (Fig. 4b, blue line) designed for this purpose following Førland et al.^55^, while change-points (Fig. 4b, red arrows) were found applying the double-shift Standard Normal Homogeneity Test (SNHT) developed by Alexandersson^56^.

We discuss the effects of flooding processes in relation to estimated past environmental changes, starting from the pulsed storm conditions during the central part of the Little Ice Age (LIA, ~ 1300–1850 CE^57^), here meaning the period 1582–1715, through the calmer weather conditions at the end of the LIA, here meaning the period 1716–1850, to the recent warming phase, here meaning from 1851 to today.

The Italian National Institute of Statistics (ISTAT, https://www.istat.it/en) reveals (http://dati.istat.it/FILE_LINK_ALBERO/FORESTE_O_SILVICOLTURA_2001_2015.zip) that over 50% of the regional territory is comprised of wooded land. This includes approximately 16% high forest, 36% coppice and the remaining portion is characterised by residual maquis. However, it is useful to start from the historical evolution of the landscape conditions to find out from which agricultural and geomorphological context the ELA area originates. During the seventeenth century (central part of the LIA), thanks to an opposite expansion of the landscape from fruit-bearing chestnut groves to wild forest for charcoal production, the heaviest floods occurred only occasionally at that time. According to the Ligurian ironworks owner Domenico Gaetano Pizzorno (1725–1775), as reported by Baraldi^58^, there is in fact a tendency that opposes conversion to wild forest, as chestnut groves provided the opportunity to maintain complex land arrangements for drainage and rainwater collection, hoeing, earthing up, fertilising and clearing the undergrowth. But Pizzorno recalls many other trees, with great competence and giving us the opportunity to reconnect with a past we no longer know anything about. Walnuts, lindens and beeches were planted to stabilise the soil and shelter the roofs from storms.

On the contrary, for less severe events, there are historical records of several floods between the 17th and late eighteenth century. In 1723, the Bishop of Brugnato, Monsignor Niccolò Leopoldo Lomellini (died 1754), criticised the management of the local hospital. He lashed them by reporting that the *Most High had already punished them by allowing the Vara River and the Cravegnola* (La Spezia Province) *to invade and devastate all the plain in the place of Brugnato, as they admitted themselves* (as reported by Piana et al.^59^, p. 215).

There are records of destructive annual floods in the area in the 1799 *Inchiesta* written by the Borghetto priest Angelo Maria Fontanabuona. He refers to a flood that occurred in the seventeenth century, which left Borghetto partially submerged and killed half of the inhabitants of the village *located on a peninsula between the Vara River and the Pogliaschina Torrent, both of them flooding the village more than once every year: in some cases water reaches the first floors of the buildings and this happens mainly in September, October and November* (by Piana et al.^59^, p. 215).

During the late eighteenth century, land use must not have been very different from that depicted in a large painting by the late-Baroque Italian painter Alessandro Magnasco (1667–1749). The painting, entitled *The Bisagno valley seen from Villa Giustinani Cambiaso*, reveals a rural landscape, dated *c.* 1740. Despite some considerable events, such as the intensification of floods in the years 1702 and 1705, it appears that, overall, floods were less frequent in the eighteenth century than in the seventeenth century (Fig. 4b, blue line to compare the first part of the end of the LIA with the central part of the LIA). In fact, the eighteenth century seem to have been characterised more by drought than by abundant rainfall^60^.

In the late 18th and early nineteenth centuries (late LIA), floods began to intensify, as evidenced by the first change-point in 1787. This was also documented in several books of the time, in which historians had noted the recurrence of floods. So writes the nineteenth century Italian writer Luigi De Bartolomeis on the province of Genoa (De Bartolomeis^61^, p. 1524):

*What remains is the province intersected by the river Entella, swollen by the tributaries Lavagna, Sturla, Graveglia, and the torrents Boate, Gromolo, Petornia o Petronia, and Crovana, […]. But all have a rapid course, and cause considerable damage to the surrounding lands due to frequent flooding […]* [our translation]*.*

Furthermore, the writings of Gianmaria Piccone from the late eighteenth century show that the territory was at the mercy of the waters and a victim of speculation in the indiscriminate exploitation of timber^62^. An accurate description of the damage caused by the frequent floods, in relation to the orographic and hydrographic characteristics of Liguria, can be found in the essay by Bertolotti^63^, entitled *Viaggio nella Liguria marittima* (pp. 19–20):

*The Ligurian torrents [...] swell abruptly and disproportionately due to the rain that falls in the mountains; they rush down, ruinous, sometimes unexpectedly, sweeping away pebbles and even large boulders, flooding and thus raising their beds. They cause great damage with their sudden floods [...]. It only takes a few hours of rain for them to overflow [...]* [our translation]*.*

**Figure 5:**
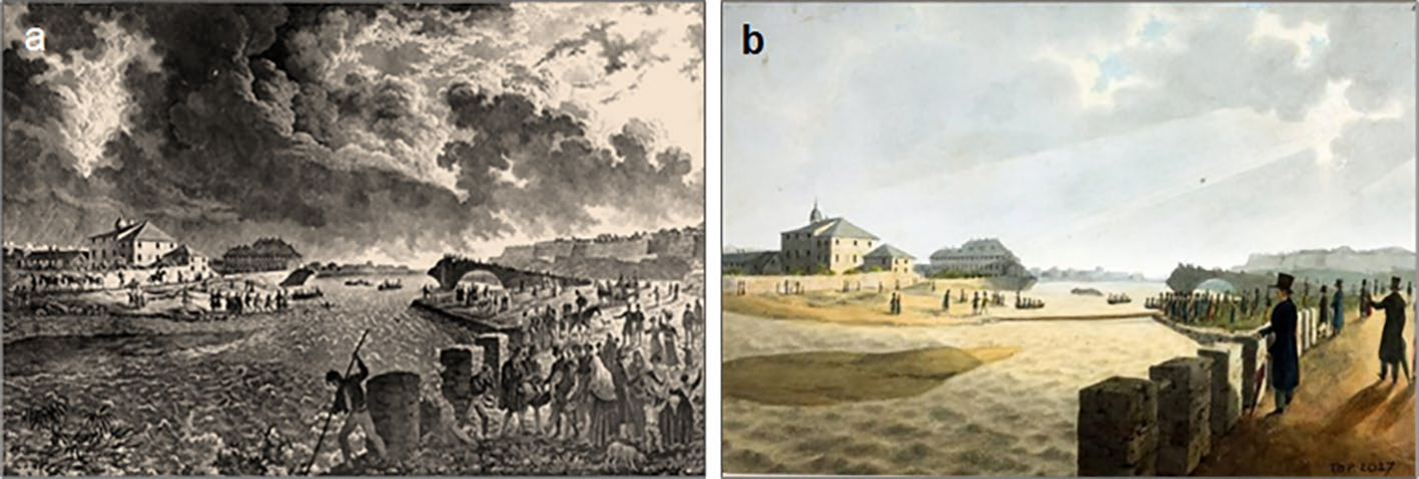
The city of Genoa at the beginning nineteenth century. (**a**), (**b**) Views of the Pila Bridge after the flood of 26 October 1822, etching and watercolour, respectively, kept at the *Centro DocSAI—Collezione Topografica del Comune di Genoa* (from: Musei di Strada Nuova—Palazzo Bianco, Genova, Italy, Uni.GE.life: https://life.unige.it/1822-2022-inondazione-bisagno).

Bertolotti’s words came a dozen years after the memorable and extraordinary flood of October 25, 1822, following the heavy rains that began on the night of 24 October (Fig. 5a,b), and continued uninterruptedly for 15 h with thunder and lightning, as reported in the Gazzetta di Genova of October 30, 1822 (Rosso^40^, p. 37):

*The rain started on Thursday evening and continued for fifteen consecutive hours in a very intense manner. Friday morning [...] at eleven o'clock everything was under water and the wave continued to rise. As the afternoon approached [...] the flood gained the entire vast plain of the Bisagno, which appeared like a muddy lagoon, from which only the tops of trees and houses emerged, submerged up to the second floor. [...]* [our translation]*.*

The weather data of the time, although measured with approximation by Professor Pagani in the area of the modern Marassi district, which he made available to the *Gazzetta di Genova*, speak of 30 inches (about 820 mm) of rain in one day^40^. Interestingly, this amount of rain is almost the same as that recorded in the violent storm (884 mm of rain) that affected the village of Rossiglione in the Ligurian Apennines on October 4, 2021^64^.

**Figure 6:**
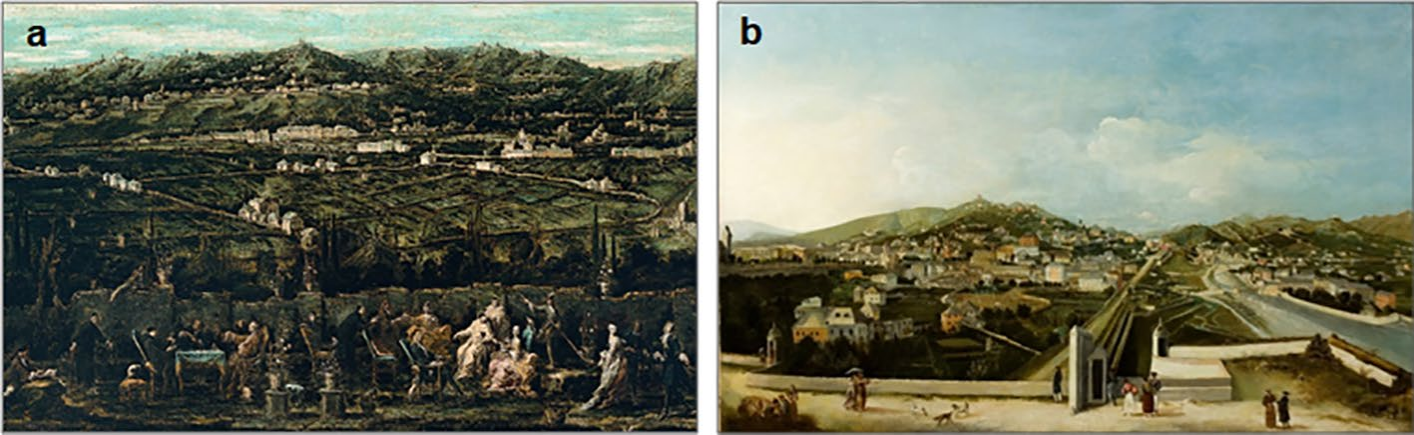
Landscape of Genoa in the eighteenth and nineteenth centuries. (**a**) View of the Bisagno plain depicting a panorama of Genoa in 1740 by the Italian painter Alessandro Magnasco (1667–1749), from Centro DocSAI—Collezione Topografica, Musei di Strada Nuova—Palazzo Bianco, Genova, Italy (https://www.museidigenova.it/it/trattenimento-un-giardino-di-albaro-circa-1740); (**b**) The esplanade of Bisagno, with Genoa in the background seen from the walls of Santa Chiara in 1834, as portrayed by the Italian painter Tomaso Castello (1792–1845), from Centro DocSAI—Musei di Strada Nuova—Palazzo Bianco, Genova, Italy (https://www.museidigenova.it/it/genova-vista-dalle-mura-di-santa-chiara).

After these first turbulent years of the early nineteenth century, the floods of the ELA became temporarily more contained, and in the middle of this century we can recall the description of the Italian abbot and historian Goffredo Casalis (1781–1856) in his geographical dictionary of 1849, which is not much different from that of the Italian Catholic bishop, linguist and geographer Agostino Giustiniani (1470–1536) in his *Castigatissimi Annali di Genova* three centuries earlier^40^. The eighteenth and nineteenth century Genoese painters Alessandro Magnasco (Fig. 6a) and Tomaso Castello (Fig. 6b) also give us back a picture of the landscapes of their time, depicting the Bisagno esplanade with Genoa, as Giustiniani had seen them.

Even at the beginning of the twentieth century, the landscape of the Bisagno Valley did not differ much from that described by 19th-century travellers in Liguria^40^, and this is consistent with the urban coverage that has not yet undergone a major change in the course of the nineteenth century (Fig. 4a, black line). During this century, however, land use changed drastically, especially in the small valley basins and, obviously, in the city of Genoa itself. Historical maps provide us with a picture of the urbanisation of the municipality of Rapallo^65^, which can be taken as representative of the ELA^40^. In this period, in fact, we can observe from Fig. 4a (black line), an exponential increase in the percentage of urban coverage from 5% in 1960 to 18% in 1980.

At the same time, there was an increase in the intensification of flooding. With the year 1967 the second change-point is reached, as probably at the same time there was also a change in the spatio-temporal pattern of extreme events. Local trends in extreme events are specific of certain areas and significant for certain durations. These spatially dependent spatio-temporal scales act on highly extreme rainfall and, in turn, on the intensification of local extraordinary flood events. This is in agreement with Faccini et al.^66^, who found that changes in rainfall regimes and human influences on the environment drive the intensification of flash-floods in the Liguria region. In particular, Genoa^39^, the Bisagno River^41^ and the village of Chiavari^67^ appear to be experiencing more frequent and intense flash-floods than in the past. Furthermore, the hourly pluviometric regime shows a positive trend, and climate data in general corroborate the occurrence of increasingly intense rainfall over since *c*. 1970, and this corresponds with the recent increase in the number of flash-floods^68^.

As pointed out by Blöschl et al.^69^, across most of Europe, the current (post-1990) flood-rich period has been much warmer than the previous flood-rich periods documented since 1500 CE, many of which instead occurred during cooler than usual phases. Recently, Wilhelm et al.^70^ found that in some small alpine catchments affected by local intensification of extreme rainfall, extreme flooding may increase with warming. This is because both short- and long-duration rainfall extremes are intensifying with warming at a rate consistent with increasing atmospheric moisture^71^.

Other studies highlight peaks of flooding or land degradation at earlier times (i.e., during the LIA) in areas of central-northern Italy close to the ELA (e.g., in the Po River Basin^10^ or in the Arno River Basin^72^). However, comparison with the ELA is difficult, in the absence of urbanised and built-up hotspots like Genoa, where not merely land-use changes, but high levels of urbanisation, have tended to have an increasingly distinct and pervasive influence on hydrological processes and landscape responses. For this reason, the methodology adopted and the results of this study offer unique insights.

## Methods

### Hydrological data

The distribution of monthly mean rainfall values (and rainy days) shows two maxima, a main one in autumn (October with 184 mm) and a secondary one in spring (April with 100 mm), and two minima, of which the main one in summer (July with 35 mm) and the secondary one in winter (February with 62 mm) (Fig. 2d). High-intensity rainfall events are mainly determined by the orographic effect, but not secondarily by the effect induced by the extensive contact between the Ligurian Sea and the mainland which, due to the different seasonal temperature of the sea surface compared to that of the ground, generates thermodynamic instability, with consequences on the genesis of cloud formations with a marked vertical development, at the origin of thunderstorms^44^. Studies indicate that a maximum of storm activity is observed over the sea near the coast in winter, in the coastal strip in autumn, and inland in spring and summer, a few tens of km from the sea^73^.

The mountain arc that meanders from the Ligurian Alps to the Apuan Alps presents a high hydrologic hazard, as it is often affected by phenomena of exceptional intensity, especially from the central sector of Genoa and in the far east, with rainfall exceeding 500 mm in a few hours^74^. The city of Genoa and its hinterland can be affected by high hourly and daily rains^75,76^, as well as the area of Mount Cappellino (650 ma.s.l.), located in the Polcevera hydrographic basin. Floods, counted in the recent period 1850–2022, are low in winter and non-existent in mid-spring (April and May), increase slowly in mid-summer and then exponentially with the onset of autumn, with the maximum number of events in October, a slight decline in November and a drop to zero in December (Fig. 2d, blue histogram).

### Documentary sources

Historical data, including from diaries, can be used independently as sources of quantitative weather, social, cultural and economic information, providing data on social vulnerability to climate extremes and allowing direct comparison with modern climatology^77^. However, this type of research is methodologically difficult and necessarily involves an interdisciplinary approach^78^, relying on collaboration between historians, geographers and climatologists^24,35,79^. Disasters of this magnitude to the detriment of cities left a lasting impression on the population of the time^80^. In many sources, the information is reported in various compound locutions for the term storm (e.g., *inondazione, tempesta, pioggia dirotta, sterminate piogge, escrescenza, fiumane, rottura di acque*).

Most research on historical climatology in Europe focuses on the early modern period (*c.* 1500–1800), due to the lack of documentary information from earlier periods for most of the region^24^. Historical climatology researchers have developed many of the methods and routines that have become crucial criteria in the field^81^. Keeping weather diaries, for instance, became a scientific practice in Europe in the late fifteenth century, in the early modern period^24,82^. Although many of these works are difficult to find, the information they convey has been recovered from more modern authors such as the Italian scholar Giovanni Battista Canobbio (1791–1853)^83^ and from the Catalogue of the CNR-AVI Project of the *Gruppo Nazionale per la Difesa dalle Catastrofi Idrogeologiche* (http://sici.irpi.cnr.it). There is also a wide range of descriptive texts whose information can only be found in generic historical sources, such as broad portrays of political, medical and religious costumes rooted over many centuries^84^.

## Supplementary Information

Supplementary Table S1.

Supplementary Information.

## Data Availability

The copyrighted material included in this article (Figs. 1, 5 and 6) has been used with permission from the original copyright owner (municipality of Genoa). The authorisation to use this material has been granted in writing (protocol no. 128006) and is available upon request (direzionemarketingeturismo@comune.genova.it). All of the data employed in this study are publicly available. The authors created the graphics labelled as a, b and c in Fig. 2 using the primary sources. The data that were generated and scrutinised in this study, including the number of floods per year and the Annual Flood Intensification Index, are accessible through a data file that has been released together with this article (Supplementary Table S1). Furthermore, the original Italian text of the referenced excerpts is obtainable through a text file that has also been published in tandem with this article (Appendix A1).
